# Evo-Devo Strategies for Generative Architecture: Colour-Based Patterns in Polygon Meshes

**DOI:** 10.3390/biomimetics5020023

**Published:** 2020-05-22

**Authors:** Diego Navarro-Mateu, Ana Cocho-Bermejo

**Affiliations:** School of Architecture, Universitat Internacional de Catalunya, 08017 Barcelona, Spain

**Keywords:** genetic algorithms, evo-devo, colourimetry, patterns, proto-architecture, generative design, emergence, flow-based programming

## Abstract

Parametric design in architecture is often pigeonholed by its own definition and computational complexity. This article explores the generative capacity to integrate patterns and flows analogous to evolutionary developmental biology (Evo-Devo) strategies to develop emergent proto-architecture. Through the use of coloured patterns (genotype) and the modification of polygonal meshes (phenotype), a methodological proposal is achieved that is flexible to changes and personalization, computationally efficient, and includes a wide range of typologies. Both the process and the result are oriented towards computational lightness for a future and better integration of the workflow in genetic algorithms. Flow-based programming is used to replicate genetic properties such as multifunctionality, repeatability and interchangeability. The results reinforce the biological strategies against other more computationally abstract ones and successfully execute the parallels of universal mechanisms in Evo-Devo that are present in life.

## 1. Introduction

One of the problems associated with generative architecture design is the rigidity of the code that defines it. As it gains in complexity and detail, its excessive parameterisation of the design increasingly leads the design towards a specific point, thus losing the exploratory character that should be associated with generative design.

One of the aspects that favours this situation is that the relationships between the elements are usually fixed and the variables that define them are those parameterised. The basis of the design is, therefore, relatively well established. Parameterisation becomes a gradient of numbers in which relationships are rarely exchanged within the same design.

The progressive complexity of the design does not contribute to making changes either, since any alteration could “break” the code.

These reasons lead us to favour a generative design based on principles such as the ones that rule evolutionary development biology (Evo-Devo), capable of generating a wide variety of individuals through rather flexible and simple rule sets.

### 1.1. Biomimetic Architecture

Architects are generally comfortable using metaphors or analogies to conduct the project, in order to provide coherence to the design.

In 1957, Otto Herbert Schmitt an American engineer and biophysicist coined the term biomimetics. Since it was established as an architecture field, biomimetic architecture is defined as a philosophy that turns to nature to solve design problems and building performances further than the general thought that it draws its inspiration from nature merely for the aesthetics of buildings.

Because natural systems offer design strategies to increase performance and effectiveness with an extensive formal repertoire, incorporating multi-faceted biomimetic principles adds considerable value to the design process [1].

In fact, the idea of nature in architecture has a long-standing tradition in history. Joseph Paxton’s 1851 Crystal Palace, for instance, was inspired by the structure of a water lily pad. Eiffel’s tower was based on the structure of trabecular struts in the head of the human femur, or on the taper of a tulip stem [2]. In 1936, Frank Lloyd Wright likened the columns in the Johnson Wax building to water lily pads and, which, although being the building’s most spectacular feature, they have nothing in common with lily leaves functionally speaking.

Other examples that became famous for their aesthetics with a nature-inspired approach, were Cecil Balmond and Toyo Ito’s design for the Serpentine Pavilion in London (2002) and Daniel Libeskind’s patterned skin based on fractals of the extension for the Victoria and Albert Museum addition in London as early as in 1996.

African architect Mick Pearce, inspired by the characteristics of termite nests, constructed the Eastgate Centre, the world’s first all-natural cooling structure, in Zimbabwe’s capital in 1996. It functions in a manner similar to how a termite nest ventilates [3].

Janine Benyus in her 1997 book “Biomimicry: Innovation Inspired by Nature” [4] popularized the term biomimicry that began to appear in literary works in the 1960s. Biomimicry can be related to nature in three ways:

- Mimicking nature form or function
- Mimicking natural processes
- Mimicking natural systems

Julian Vincent is one of the main references in researching technology applications developing the idea of adaptive designs based on biology as the basis for advanced architecture. For him, nature and the organisation of biology and engineering are very different: organisms develop through a process of evolution and natural selection; biology is largely descriptive and creates classifications, whereas engineering is the result of decision-making. 

Vincent also believes that the transfer of a concept or mechanism from living to non-living systems is not trivial. A simple and direct replica of the biological prototype is rarely successful [5].

For that very same reason, the design metaphor has been kept abstract, focusing on the process that may later be classified according to the Khoshtinat scheme, where biomimicry works on three levels [6]: the organism, behaviour, and ecosystem; each one with subgroups based on form, material, construction, process and function. The process explained in this paper belongs to the “behaviour level–process”:

*“The building works in the same way as a termite mound would; by careful orientation, shape, materials selection and natural ventilation for example, or it mimics how termites work together.”*

[6].

In 2008, Kolarevic presented his famous “Post-Digital Architecture: Towards Integrative Design”. In his research he proposed the idea of integrative design, (in opposition to integrated design), in which methods, processes, and techniques are discovered, appropriated, adapted, and altered from “elsewhere,” and often “digitally” pursued [7].

As he mentioned, collaborations with mathematicians were increasingly sought out to embrace mathematics as a source of inspiration not only for aesthetic reasons but also to investigate new ideas for the behaviour of architectural structures.

By then biomimicry was already a source of inspiration for innovation stating that designing a building to visually resemble a natural model was less advantageous in many cases than to design a built system that behaves like a natural model. With these new sources of inspiration Kolarevic mentions that algorithmic thinking, biomimicry, computation, digital fabrication, material exploration, and/or performance analyses are necessary to “discover and create a process, technique, or a product that is qualitatively new.” He considered scientific and engineering ideas as starting points for design investigation.

Finally, Erdines’s proposal for biomimetic strategies applied in tower design [8], put forward a distributed intelligence based on object oriented programming where biomimetic analogies formulate the basis of the geometric behaviour mechanisms for the structure together with motion behaviour mechanisms and establish the parameters for the process.

Her project structure based on the idea of integrating the properties of natural branched constructions properties employs real-time generative form-finding techniques, described as bottom-up processes where design output emerges from the interaction between autonomous agents and their environ ment in the object-oriented programming environment.

The aforementioned research stablishes a background for the design philosophy of this paper, in which architecture design advances toward processes and behaviour strategies founded on biolearning, and digital tools become necessary to simulate the concepts and advantages of the extensive biological repertoire.

### 1.2. Flow-Based Programming as Methodology

To address the purposes of this article, we suggest the use of flow-based programming (FBP) as a methodology to manage the stream of information and the generation of digital prototypes. FBP approaches information management in a more organic and flexible way, which brings it closer to the bottom-up systems of biology and emergency.

*“It views an application not as a single, sequential process, which starts at a point in time, and then does one thing at a time until it is finished, but as a network of asynchronous processes communicating by means of streams of structured data chunks, called “information packets (IPs).”*

[9].

As opposed to the linearity or sequentiality of traditional scripting [10], FBP easily handles multi-processes (parallel execution), which are definitely more typical of organic or complex systems. FBP also facilitates the exchange of information packages and interoperability. That is, the multifunctionality of different parts as happens with the gene code. These processing blocks are also known as components, IPs (information packages), software black boxes, or data chunks [11].

These IPs can be understood as genetic tools capable of processing and transforming genotype data into a virtual phenotype. 

FBP has a clearer structure, where the interface itself is the relationship of the data. This makes it easier to read, learn and debug. These may be reasons why, despite not being a new approach, PBF has proven to be on the rise in recent years, e.g., NoFlo, UnReal Blueprints or LabVIEW [12]. Therefore, it is important to establish appropriate flows that can be replicated by similar software. For that reason, this article describes the steps and relationships established one by one.

The authors understand how the digital momentum within the architectural field is due to its accessibility through visual programming or algorithm-aided design (AAD) [13]. The impact of these types of software and easy-to-use tools should be assessed to develop a critical mass and discuss both the designer’s new abilities and the constrains of non-mathematical experts.

To accentuate the previously established relationships, a methodology based on AAD has been created through the use of Grasshopper software (node based visual programming plug-in for Rhinoceros 3D, complex surface modelling), which shares many of the virtues of FBP, as well as other elements that make it especially relevant.

## 2. Case Study: Approaching Evolutionary Development Biology (Evo-Devo) Algorithms

In the 1990s, a series of conferences on evolutionary algorithms (EAs) established a common background for those outside the computational field, mainly designers, engineers, and architects. 

Evolutionary algorithms allow designers to solve problems that are yet to (or cannot) be defined. Their capacity to evaluate large amounts of data, without understanding the connection between the elements enables designers to tackle dynamic situations with non-deterministic contexts or environments with unobservable variables [14].

This provides a new approximation towards biology simulation, where designers must be concerned with how things ought to be, instead of how things are [15].

Most notable contributions to the relation between EAs and architecture have been led by John Frazer [16] because of his interest in environment analysis for the development of responsive architecture. Frazer stated that architects should evolve rules (relations) for generating form, rather than the forms themselves; dealing with the hypothesis that, hierarchical levels should be prioritized over defined and complex relations.

These strategies were later further developed by Michael Weinstock [17], who would recognise and adopt Evo-Devo strategies and is considered the initiator of the experiments presented in this document.

*“It is precisely the notion of loose control that can be postulated as a possible new authorial model, pitted against the totalizing normativity of current computational design methods.”*

[18]

However, computation has failed to adapt major breakpoints in evolutionary biology such as the incorporation of developmental (embryonic) science. 

**Figure 1:**
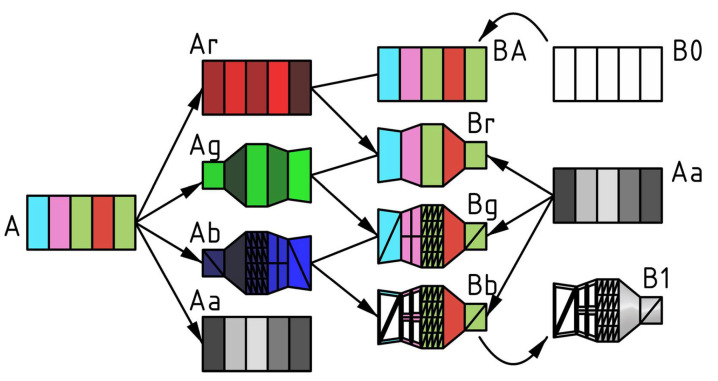
Diagram inspired by the representation of embryos in 1995 Physiology or Medicine Nobel Prize press release [21]. Diagram showing colour-mesh development proposed by the authors. A = genotype and its channels. B = phenotype and its modifications. Lower case: r = red, g = green, b = blue, a = alpha. Numbers: 0 = original body plan, 1 = final phenotype.

Research concerning the understanding of genetics and its functioning has left brilliant discoveries during the past decades such as Jacob’s genetic regulatory mechanisms [19] or the existence of genes regulating early embryonic development by Nüsslein-Volhard and Wieschaus (Nobel Prize 1995) (Figure 1) [20].

These discoveries have led to the incorporation of embryonic development into the theory of evolution, integrating new mechanisms that stand out for their simplicity but are, nevertheless, generators of great diversity [22]. It is evident now that biologic development is ruled by universal rules on simple geometries where genetic switches (patterns) control homeotic genes (shape) [23,24].

Relevant mechanism for geometry generation such as the ones described in Evo-Devo have not been put on common ground and no software has built them in.

The premise of this article is to further develop the conversion of biological strategies into computational processes that can expand capabilities within the field of design and particularly in architecture. Among Evo-Devo mechanisms, the authors find the following points especially valuable:

- The construction of a free and random body plan.
- The integration of colour patterns as genetic data or switches.
- Allometric deformations to disturb topological premises.
- Topology modification on polygonal meshes as homeotic genes or allometric deformation.

### 2.1. Body Plan Approximations

Since the very beginning, a body plan has been established as a white canvas on which to apply subsequent modifications. Evo-Devo properties should be later applicable to any body plan and for this reason generating a body plan is kept as an isolated event. Moreover, the authors believe that the use of the same homeotic changes through switches on the body plan itself could give rise to fractal—and therefore redundant—structures.

Constrains regarding the body plan have been limited to a 4-cell sided cubical grid (64 cells), meaning that any combination inside that grid is possible: i.e., 4X1X1, 1X2X3 or 4X4X4. Therefore, the initial segmentation of the ‘embryo’ is defined as the creation of the body plan inside the aforementioned grid, where the existence of points inside a cell define their existence (an empty cell with no point will be void in the body plan). A simple voxelization algorithm is used to build continuous meshes which detect and delete inner faces [25]. In the case study, the “voxel mesh” component by Mateusz Zwierzycki was used.

Three approaches were used to address this first level in the body plan segmentation.

1. Completely random generations based on the point population of the spatial grid.
  a. Input 1: number of points populating the grid (1-64).
  b. Input 2: seed of the random algorithm that generates the number of points.
2. Auxiliar linear connections to force continuity between the cells.
  a. Input 1: number of points populating the grid (reduced by half, 1-32).
  b. Input 2: seed of the random algorithm that generates the number of points.
  c. Input 3: proximity curve component.
3. Patterns built from random seeds.
  a. Input 1: Random pattern A.
  b. Input 2: Random pattern B.

**Figure 2:**
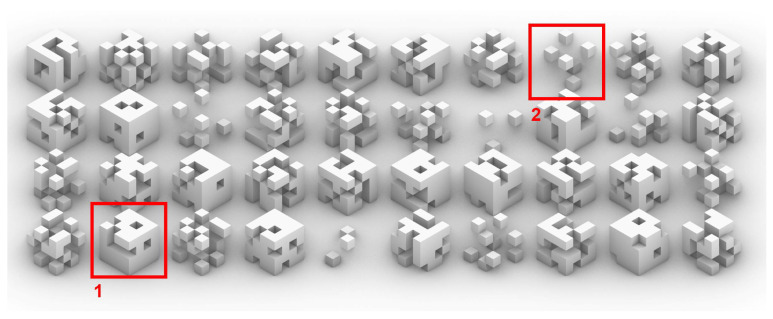
Results range from massive cubes with holes (1) to floating detached voxels (2).

It is obvious from the beginning that the type-1 body plan (Figure 2) quickly develops rich and complex results. This type of random algorithm is always well received in the design field. However, a general look at the overall population of individuals generated shows similar characteristics and a homogeneous population. 

**Figure 3:**
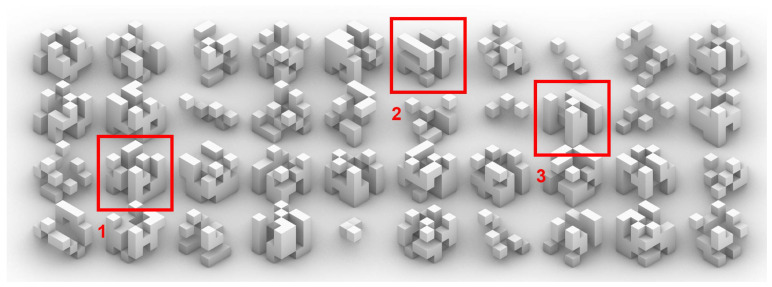
An increase in long straight arrangements, both vertical (1 and 3) and horizontal (2), can be observed because of the connectivity component.

The type-2 body plan (Figure 3) adds geometric relationships to the random populations in order to establish more complex relationships. By using proximity algorithms, new cells in the spatial grid are activated, connecting distant cells with linear cell repetitions. In order to compensate this, the initial population maximum was reduced to half (1–32). The objective is to be able to establish hierarchies and distinctive properties from the very beginning of the body plan’s construction.

**Figure 4:**
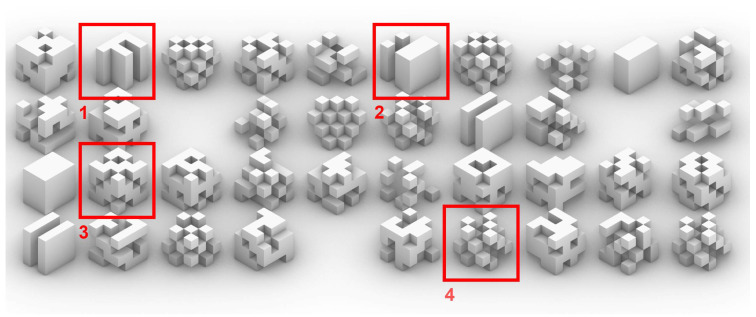
The chosen elements show vertical structures (some of them separated like towers) (1 and 2), symmetric (3), or repetitive patterns (4).

The type-3 body plan (Figure 4) is introduced later on in the experiment, after observing positive results in later steps stages of the case study. Despite maintaining a different source, the body plan is generated through a Boolean pattern that is repeated throughout the spatial grid.

This repetition together with the defined boundaries of the spatial grid will genetrate distinguishable patterns and structures far more often than previous body-plan types [26].

### 2.2. Emergence, Patterns and Colourimetry

The dynamic generation of patterns has numerous advantages for generative design. To begin with, the very same generation becomes a very flexible, always changing, value that can have many and different impacts on the design. A second advantage is the computational lightness of colour-data patterns, which in this case allows the transmission of information throughout the development process in a very efficient way. Finally, it has an ability to emerge uninvolved structures through simple rules.

As can be seen in the protein system for DNA, some colour space systems could perform a task of combination and regulation of information. Complexity science has established that the environment can be understood through dynamic interconnected networks where smaller and direct relations are constantly rearranged and produce spontaneous behaviour like climate, chemical systems, biological networks or even market dynamics [27]. In this regard, Wolfram’s work [28] establishes simulations where simple digital rules manage to replicate patterns similar to those present in nature. Alan Turing also manages to simulate model biological patterns on more simpler chemical reactions that can easily be simulated digitally [29].

Patterns and colours fulfil multiple functions in the biological world such as camouflage, physical response, sexual selection, signalling or mimicry; they can represent the underlying structure of morphogenesis such as spirals, L-systems, waves, tessellations, bubbles, cracks, spots and many more [30].

However, both researchers and designer should keep human brain ability in mind to understand and relate shapes, colours and patterns. The colours of the visible spectrum, for example, are not equally distinguished by human thought from the visual spectrum [31], and finding patterns in chaos is also a subjective quality [26]. 

Previous research studies have shown the functionality of 3D polygon meshes to mimic biological body plan structures [32]: patterns, Boolean operations, subdivision, orientation, symmetry, and polarity. The very same structures observed in embryology are often used in 3D modeling software. For instance, subdivision—like tessellation—can represent from cell aggregation to maxels [33], a hierarchical strategy that is fundamental to any complex system. 

In contrast to most vector geometries, meshes have the quality of embedding colour data in their vertices. While most geometry data are referred and depend on their own geometry, colours can carry on data on through topological changes without being altered, becoming a great source of data transmission [34].

The mathematics of colourimetry enable us to use colour as numeric values, represented by several channels that can express different properties beyond the mere fact of expressing colourful patterns. Most colour spaces are composed of three channels (RGB: red-green-blue, LAB: lightness-green to red-blue to yellow, HSB: hue-saturation-brightness) plus the alpha one, except CMYK, that has four channels (cyan-magenta-yellow-key) and cannot carry an alpha channel.

In order to better distinguish the patterns, RGB and CMYK seem to be the best option because of split colours (instead of assigning lightness or saturation to a gradient of tones). Of those two options, RGB was chosen because seems more in line with the digital realm [35].

Therefore, the use of colour in this case study does not only refer to visualisation but also to a light method capable of transmitting data from original patterns (genes) to an expression that allows easy differentiation of its parts and offers sufficient richness. 

**Figure 5:**
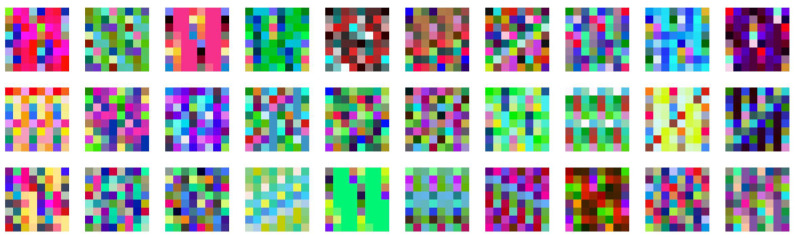
Thirty individuals showing patterns generated. This 2D grid displays 64 (8 by 8) cells, the same number of the 3D grid of the body plan (4 by 4 by 4). The selection shows interesting patterns based on their relation with the grid size, producing diagonals (4,1), one-dimensional repetitions (1,2), checked (8,2), massive (5,1) or almost random (5,3).

A pseudo-random algorithm is proposed to generate colour patterns that can work as genotypes (Figure 5). The output values generate a list of colours that will become the infinite pattern to be applied on the desired mesh:

- The pattern generator is based on two types of “random numbers” (L and S) components that are later subdivided in four more (R G B A).
- L components outputs 4 numbers within the range of 5 to 30, and their seed is controlled by the number of the individual (1 to infinite). This range was chosen to discourage the sync between the pattern and the body plan in cell side-size and cell total number (4 < 5 and 30 < 64). The value of L will become the length of each of the four colour channels, in other words, the number of numbers generated by R, G, B and A.
- S components ranges from 0 to 100 (arbitrary numbers) and is used to output new seed values for R, G, B and A. Seed from component S is also determined by the individual number.
- R, G, B and A components range from 0 to 255 (typical units for RGB colours). As exposed above, the number of colours in their channel is controlled by L, while their seed by S.

The length mismatch among the channels produce pattern intersections in such a way that there are different regulatory genes that overlap fragmentations during the development of the organism. A very common example is the overlapping of melamine genes in skin patterns, like in the case of the panther.

### 2.3. Allometry Based on Mesh Faces

**Figure 6:**
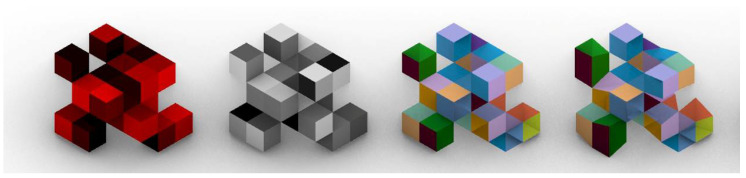
An individual with its corresponding channels. From left to right: red channel, alpha channel, original mesh and allometric mesh.

The red colour channel will be used to simulate allometry changes in the phenotype (Figure 6). Allometry refers to changes in relative dimension of the body parts, allowing it to produce different shapes without changing the topology of the body plan [36]. Frequently referred to as the fourth spatial dimension, the value of allometry changes can clearly influence architectural design through scaling spaces, proportions or geometric relationships.

As previously described, the faces of the mesh representing the body plan are divided into three groups based on their “red value”. In parallel, the strength of these modifications is based on the fourth channel (alpha), the data of which are numerically remapped to fit corresponding modifications. Alpha values have been constrained to avoid collisions with other faces.

Therefore, the red value works as a switch pattern that enables specific modifications, while alpha channels act as a booster value to enhance those genetic modifications.

In this case study, the authors have improved the mesh changes. While previous experiments applied scaling to single joined meshes, a new algorithm has been introduced to select vertices from faces that will be scaled and will, therefore, modify neighbour faces. They show a better interrelation between the body plan parts.

The changes based on red and on alpha channels are as follows:

- Group 1 (0–85). No changes.
- Group 2 (86–170). Non-uniform scaling of the face based on Z axis [0.4–1.6].
- Group 3 (171–255). Face scaling from random vertex [0.7–1].

### 2.4. Recursive Fragmentation/Subdivision

**Figure 7:**
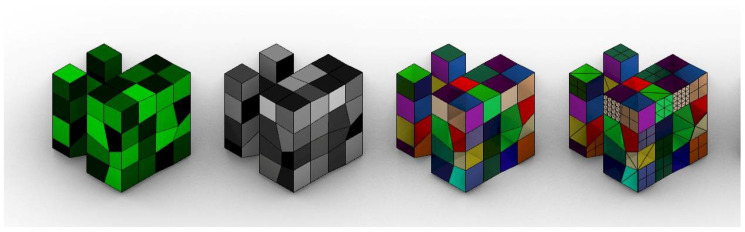
An individual with its corresponding channels. From left to right: green channel, alpha channel, original mesh and allometric mesh.

The blue colour channel simulates the fragmentation of the body plan, which in turn becomes the subdivision of the mesh that will later be affected by the homeotic genes (Figure 7). The topological subdivision of the body plan has been described as a necessary and very helpful tool in evolutionary development. The duplication of parts allows for later specialization /and multitasking, as redundancy in the organization provides new opportunities [23].

In architecture, subdivision has a direct impact on the tiling and on the size of the elements that compose that geometry. A single face with a large hole depicts a window, while 64 small holes become a sun filter. Size and quantity are always related, representing alternative ways of functioning under specific conditions and for different materials.

The changes based on green and alpha channels are as follows:

- Group 1 (0–85). No changes.
- Group 2 (86–170). Catmull-Clark Quad subdivision (0–2) [37].
- Group 3 (171–255). Triangular subdivision by Charles Loop (0–2) [38].

### 2.5. Homeotic Genes as Topology-Dependent Mesh Modifiers

**Figure 8:**
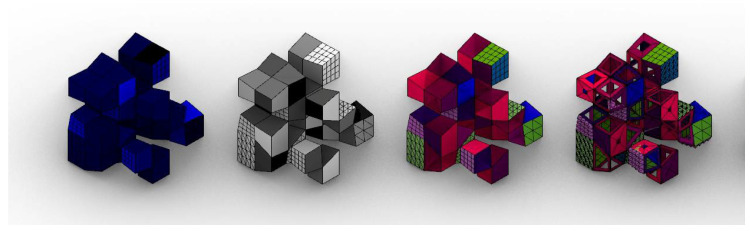
An individual with its corresponding channels. From left to right: blue channel, alpha channel, original mesh and allometric mesh.

As has been previously stated by the authors [32], meshes excel at applying different modifications in a universal way, and allow a new toolkit of genes to be introduced that alters the topology of the case at hand (Figure 8). Depending on the scale or step, these modifications can produce massive changes or can become superficial adaptations.

The third colour channel, blue, introduces three homeotic genes that express different architectural qualities such as openings, walls, or a third one made of pyramids left to interpretation (green spaces, decoration, second material…). These genes have a direct impact upon the phenotype representation. 

Again, the alpha channel is used to enhance the homeotic genes, changing the size of the openings, or the depth of the pyramids. Obviously, these mesh modifiers are influenced by the deformations of the allometric changes of the red channel and are size-dependent because of the green fragmentation channel.

## 3. Results

The final version of the algorithm, following Evo-Devo mimicking requirements, has also been consistent with the FBP philosophy and, as a result, a reduced number of IPs (information packages) was needed to build the prototype generator. The basic blocks are the following:

- Random colour pattern generation (Boolean pattern for the body plan);
- Colour deconstruction in ARGB Channels and value remapping;
- Separation of faces into three domains within a colour;
- Polygonal mesh modifiers application (allometry, fragmentation, homeobox genes).

**Figure 9:**
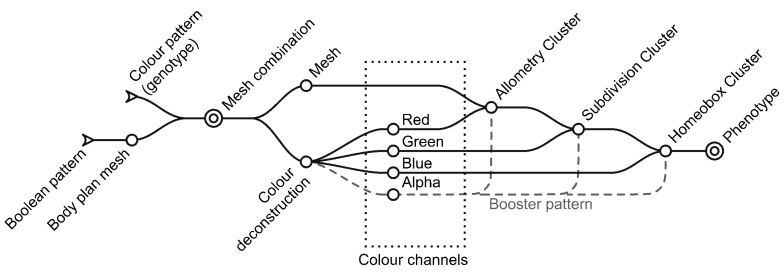
The deconstruction of pseudo-random colours and their deconstruction based on colourimetry structures allow the information embedded in the developing phenotype since the genotype generation to be carried.

Based on the case study’s virtual prototypes, the resulting attempts to establish a digital workflow that mimics Evo-Devo strategies are the following (Figure 9): 

The definition has 235 essential components, although 43 more were added for visualization and analysis purposes. Average computing time of individuals is 240 milliseconds, processed in a computer with the following specifications:

- OMEN by HP Laptop 15-dc0xxx
- Windows 10 Enterprise 64 bits
- Intel (R) Core (TM) i7-8750H CPU @ 2.20 GHz (12 CPUs)
- 16384MB RAM
- DirectX12
- NVIDIA GeForce GTX 1060 6GB

Technical results and performance conclusions will be discussed later on.

**Figure 10:**
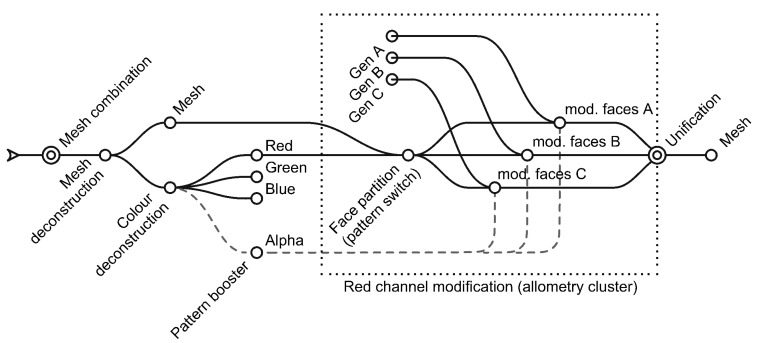
Detail of the data flow inside the red colour channel.

When zooming into any of the clusters that develop the prototypes, the partition of the mesh into faces that share specific colour ranges (Figure 10) can be observed. At some point, the faces are also distributed based on their colour, changes based on the gene related, enhanced by the fourth channel (alpha) and, finally, reassembled prior to the next partition in the following step of development.

**Figure 11:**
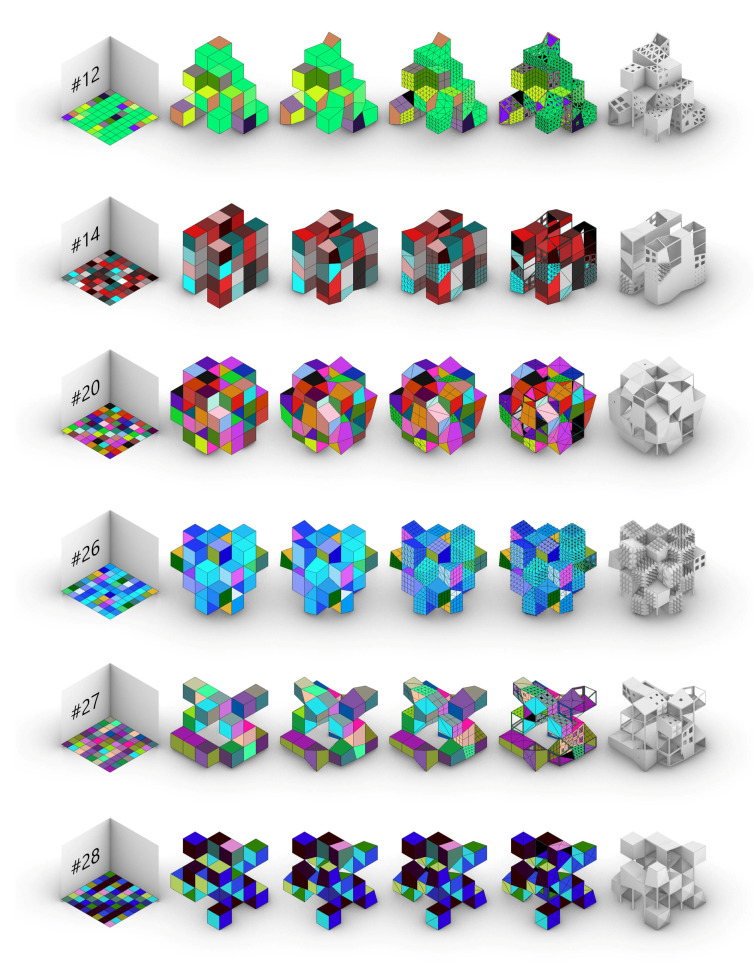
The most singular individuals within the generative algorithm. Among them: pyramid, parallel blocks, pseudosphere, symmetric density, random openness, complex minimal, towers group, massive perforated, twisted towers, symmetric minimal, block with towers, and isolated cubes.

**Figure 12:**
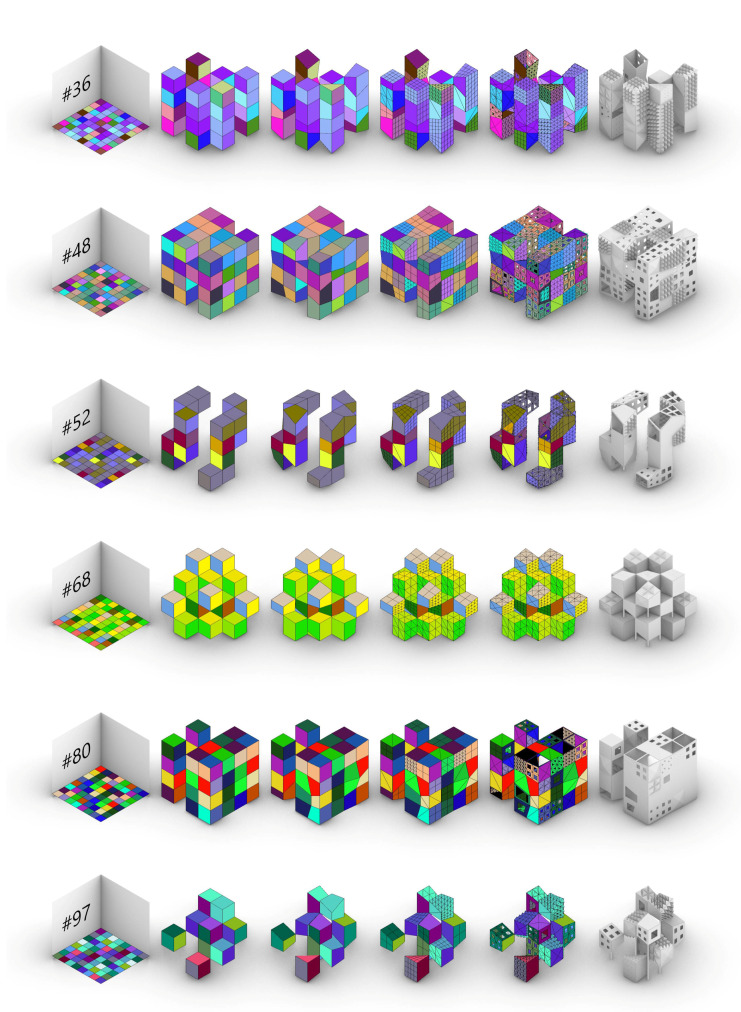
The most singular individuals within the generative algorithm. Among them: pyramid, parallel blocks, pseudosphere, symmetric density, random openness, complex minimal, towers group, massive perforated, twisted towers, symmetric minimal, block with towers, and isolated cubes.

Some of the most singular and interesting individuals, 12 in all, have been chosen among the first 100 individuals. They represent the great variety achievable by the algorithm, proof of the colossal design space developed, with millions of possible combinations (Figure 11 and Figure 12).

As known, the impact of the initial body plan is a powerful tool that defines most of the typology of the building. However, the successive changes all along the Evo-Devo strategy can completely bend that typology into empty structures, baroque façades, minimal volumes or twisted geometries.

## 4. Discussion and Analysis

**Figure 13:**
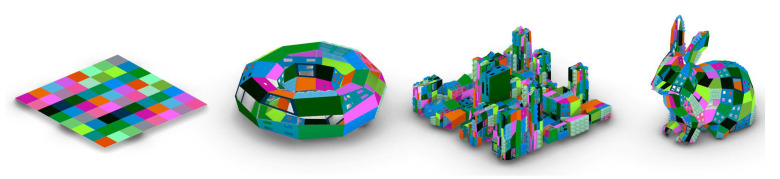
Example of the algorithm’s flexibility: colour pattern genotype converted into three different phenotypes based on their initial body plan/mesh (torus, city, rabbit).

After much polishing and optimising, the data flow was reduced to 235 components, implying a rather small definition. However, it is better than the reduced number of components to have the possibility to cluster them into packages with simple data types (input-output of meshes with embedded colour channels) following the FBP strategy. This conveys the Evo-Devo philosophy, enabling genetic properties such as multifunctionality, repeatability and interchangeability (Figure 13).

Regarding performance, 240 milliseconds is a remarkably good value. Further optimisation of genetic algorithms would easily require around 500 individuals per population in a 100-generations run. In total, that would be 50,000 individuals which will take 5.5 hours to calculate, a reasonable value considering the means and the variety possible. Previous experiments addressing evolutionary simulations in architectural design quickly escalated into several seconds, even though they were simpler geometrical models, increasing evolving time up to 6 times.

**Figure 14:**
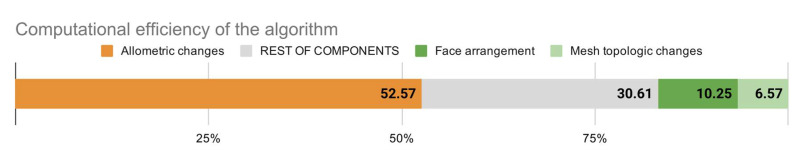
Percentages of computation usage in descending order. Average of 100 individuals (4-cells sided cubes).

This is one of the main reasons to develop a superefficient process that is light and powerful: the need to calculate thousands of individuals inside evolutionary algorithms. In the same way, the scalability of the project is a vital factor that has proved to be efficient:

To test this, the algorithm was enlarged to different sizes and tested on 100 individuals to calculate average computing time (Figure 14). Obviously, patterns with a tendency to subdivision and complex topology transformations are exponentially heavier than the simpler ones, thus a large increase in the computation time was expected. However, the data showed that the increase was relatively small considering the exponential character of the geometry.

Analysing the computation time of each of the parts of the algorithm, 3 stand out above the overall set.

- What was originally estimated to be the heaviest burden (the generation of meshes from homeotic genes) turned out to have experienced the smallest of the three (6.57%).
- The second heaviest is the calculation, subdivision, and organization of the meshes according to colourimetry. Although it is heavy, it is understood to be the most demanding data operation and its values are of no concern (10.25%).
- The first position in the computational load came as a surprise. It was the reconstruction of the meshes during the allometric process. As it is an early stage in the development of the individual, and the displacement is of mere vertices, it is strange that it corresponds to 52.57% of the process of the algorithm. Undoubtedly, if a future optimisation of the algorithm should start at this point, the priority should be to find an alternative for the deformation of meshes that is more consistent with the rest of the definition.

Further recursions of the algorithm (28-cell sided cubes (28 × 28 × 28)) increased the impact of allometric changes up to 71% of the computational calculation.

As mentioned before, together with efficiency, scalability is one of the main points of the hierarchical system able to reproduce fractal geometries. Like life itself, the Evo-Devo algorithm has to adapt to different sizes and requirements (furniture, house, block or city).

To test this, and based on the discoveries about the impact of allometric changes, three individuals were chosen and then escalated to bigger sizes (Table 1). These individuals are distinguished by their content in allometric changes (red channel): average, high, and low. 

**Figure 15:**
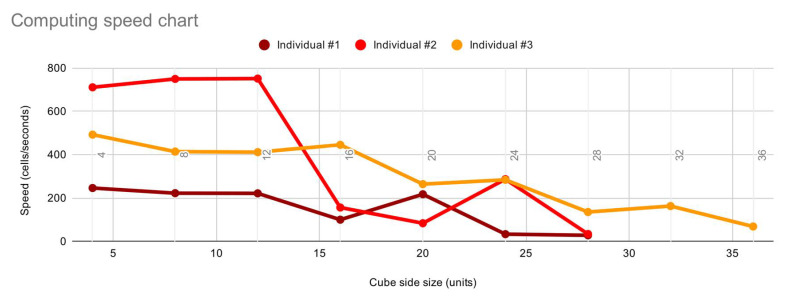
Speed calculation based on cell calculation speed. Individual #1: average allometric presence. Individual #1: high allometric presence. Individual #3: low allometric presence.

From the resulting data (Table 1) showing processing time and cube size, a graph is created to compare cell calculation speed (Figure 15). 

The discrepancy in the calculation of 4-cells and 8-cells sided cubes is due to their low computation time. The margin of error of the software and possible interferences from the operating system are bigger than the calculated times. To bypass this, calculations were carried out of 20 individuals at the same time and then the average was calculated.

The following points observed are worth mentioning:

- Speed remains constant up to 12-cell sided cubes.
- Individual #2 performs a dramatic drop due to its high allometric value (16-cell sided cube).
- Individual #2 and #3 cannot calculate beyond 28-cell sided cubes due to the allometric impact.
- Individual #3 has a lower curve thanks to the absence of allometric changes.
- During the cell sizes of 4, 8 and 12 cells other modifications like mesh creation have more impact than allometry changes.
- Depending on the pattern, discrepancies can be observed (24-cell sided cube in individual #2 and 20-cell sided cube in individual #1).
- Despite the exponential growth of the design space, the speed is reduced in a linear manner.

Beyond the revisable aspects, the lightness of the digital meshes, and the reduced number of components because of the information embedded in the colour channels, proved to be successful. While other digital strategies require separate layers of information, colourimetry on meshes enables subdividing and altering without changing the information relationship with the object. Information and form become a single identity. The authors are confident that the proposed workflow is efficient and powerful enough to work within an evolutionary algorithm.

The proto-architecture prototypes have exceeded expectations and provides an excellent starting point for researching the development and evolution of architecture based on more specific terms of the discipline such as: sunshine, area, housing density, function, connectivity, materiality or cost.

### 4.1. Design Considerations and Architecture Applications

Randomness as a design is rather homogenic. Although exceptions may arise over time, most of the designs will be a non-structured look that may seem complex but will be very similar to the rest of the random results: they do not express any structured idea behind the project.

This can be observed in Wolfram’s experiments on cellular automata, where most of the settings do not produce any relevant results [28]. Randomness can generate anything, but most of the cases are not interesting, and it thus becomes ineffective when generating designs. 

Therefore, the use of readable patterns has proved valuable for generating designs with evident differentiation and potential value to express concepts. As mentioned before, the perception of patterns should bear the following in mind: the long ones are understood as random, the short ones as repetitive, and the medium ones as structured [26].

Future experiments that incorporate this developmental process into evolutionary algorithms should avoid the random generation of patterns. Instead, a more progressive/logical approach is recommended so the algorithm can perform a better optimization search. This was not necessary in the present case study.

Lastly, when looking for variety, gradients should be taken into account. Large domain gradients with high divisions go against diversity. To obtain a few extremes, there are a lot of very similar intermediates. It greatly enlarges the design pool but does not generate new singular individuals. Making use of key points within gradients is thus recommended. For instance, 0–3–6–9, can be more efficient than 0, 1, 2, 3,…, 9.

Regarding architecture considerations, designers should differentiate two main groups: generation strategies and optimization aspects. Generation strategies will largely depend on architectural aesthetics and should be addressed through the developmental genetic tool kits: how mesh topology is changed through genes, the way the body plan is affected by the site plot, face subdivision based on material tectonics, or spatial allometric deformations to ensure program adaptation.

Meanwhile, more universal aspects or design goals should be defined as the objectives to be optimized by the evolutionary algorithm: sunlight, built area, minimum distances, energy consumption, spaces connections and relations, etc. These needs are being widely covered through different kinds of software and can easily be incorporated into the analysis and selection process of the evolutionary solver.

Selected individuals in the results of this case study have proven the great diversity that the algorithm can use to fulfill both aesthetic and functional architectural necessities. What remains to be done is to continue developing new parametric and computational relations within the architecture discipline, carefully incorporating insights from fields like shape grammar, space syntax, generative distributions, or network topologies.

### 4.2. Further Development on Mesh Patterns

Future research should implement and test different approaches for pattern growth on meshes. Developing patterns that are dependent on geometry’s topology would help to better understand explicit phenotype relations. 

On the one hand, there are patterns that grow based on the particularities of the body plan organisation. Complex situations with organic geometries or irregular meshes are worth considering. For instance, patterns always grow from corners, from the ground or on the smallest faces [39]. 

On the other hand, applying adapted “cellular automata” on topological surfaces is recommended. Instead of using two-state-cells (dead/alive), history cells could be considered as further cell types, adding time to help establish typologies and relations [40]. Useful applications of these structures can be seen in Bochenek’s work [41] where mass distribution is optimized for shape performance.

Likewise, reaction-diffusion chemical patterns proposed by Alan Turing to simulate morphogenesis in multicellular organisms would bring an extra level of depth [29]. These natural process have already been applied to both meshes [42] and in architecture [43]. Overlapping different patterns with different functionalities could generate patterns that are geometry-dependent. 

Lastly, it is recommended to improve the flexibility of overlapping patterns mixed with dynamic remeshing. This is particularly interesting when combining different urban uses or specific design elements that have different areas of impact and scales. 

### 4.3. Skipping the Genotype–Phenotype Conversion

An interesting approach as a matter of further research could be the use of data-driven methods that selectively analyse the genotypes. For the past three years, several data-driven methods following some deep learning procedures have been presented. They are not only related because of image analysis and recognition but also to because of different situations that require visual analysis, interpretation and decomposition.

Following that path, an approach to the process similar to some of the deep-learning neural networks could be interesting for further study and development.

Decomposing (interpreting) the patterns—genotypes—during the first part of the process might be a way of improving performance in analysis and re-composing the phenotypes proposed by the neural training.

Considerable improvements in performance can be observed in the example presented by Holden and others [44] in 2017 regarding velocity improvements in fluid simulation. Their method is based on an initial decomposition of the data through principal component analysis for dimensionality reduction to later train a Neural Network in a faster way.

Some methods selectively accelerate components of the physics process; for example, Thompson [45] uses a highly specialised deep neural network to solve the incompressible Euler equations of a fluid simulation. Lahner [46] combines a coarse simulation with a generative adversarial network that adds high-frequency details such as wrinkles of cloth in a game simulation. 

As for building simulations or the design dynamic game environments in real time, runtime performance is crucial in the equilibrium that must be achieved together with the computation power needed and the accuracy of the details.

Developing a technique for decomposing data from genotypes to train the process of the re-composition of the genotype–phenotype conversion could improve real-time speed allowing for greater complexity and detail. It could also increase the evaluation capacity allowing a relatively complete genotype analysis.

## Figures and Tables

**Table 1:** Successive tests were carried out until the calculation value exceeded 10 min.

Cube Side Size (Units)	Cells Space (Units)	Average Allometry	High Allometry	Low Allometry
		Individual #1 (Seconds)	Individual #2 (Seconds)	Individual #3 (Seconds)
4	64	0.26	0.09	0.13
8	512	2.30	0.68	1.24
12	1728	7.80	2.30	4.20
16	4096	40.90	26.20	9.20
20	8000	36.80	95.40	30.30
24	13,824	413.00	48.20	48.70
28	21,952	779.00	643.00	162.00
32	32,768	-	-	201.00
36	46,656	-	-	678.00
